# 

**Published:** 1896-05-23

**Authors:** 


					the HOSPITAL. ABSOLUTELY PURE therefore BEST,
May 2^rd, is9fi.
UADBURY'S ww tes-, CADBURY'S
/ COCOA W?I JF* COCOA
" The standard of highest purity at present <5?J NO ALKALIES USED,
attainable."?Lancet. <?=*-"
W/CW HOSPI TAL
No. soiV Vol. xx. MVITH EXTRA NURSING SUPPLEMENT. pP^LXW?.^?B'
r Registered ??> tae General fost Office aa a Newspaper tor Monthly Parta, 6d.; post free^Bd,
OATUBDAY, May 23rd, 1896. ?ronami^ion in the United Kintrdom and Abroad.! Ditto per Annum, 8a.6d., pogt free.

				

